# Self-Rated Health and Cardiovascular Disease Incidence: Results from a Longitudinal Population-Based Cohort in Norfolk, UK

**DOI:** 10.1371/journal.pone.0065290

**Published:** 2013-06-03

**Authors:** Rianne M. van der Linde, Nahal Mavaddat, Robert Luben, Carol Brayne, Rebecca K. Simmons, Kay Tee Khaw, Ann Louise Kinmonth

**Affiliations:** 1 Department of Public Health and Primary Care, University of Cambridge, Cambridge, Cambridgeshire, United Kingdom; 2 Medical Research Council Epidemiology Unit, University of Cambridge, Cambridge, Cambridgeshire, United Kingdom; Uppsala Clinical Research Center, Sweden

## Abstract

**Introduction:**

Self-rated health (SRH) predicts chronic disease morbidity including cardiovascular disease (CVD). In a population-based cohort, we examined the association between SRH and incident CVD and whether this association was independent of socio-demographic, clinical and behavioural participant characteristics.

**Methods:**

Population-based prospective cohort study (European Prospective Investigation of Cancer-Norfolk). 20,941 men and women aged 39–74 years without prevalent CVD attended a baseline health examination (1993–1998) and were followed for CVD events/death until March 2007 (mean 11 years). We used a Cox proportional hazards model to quantify the association between baseline SRH (reported on a four point scale – excellent, good, fair, poor) and risk of developing CVD at follow-up after adjusting for socio-demographic, clinical and behavioural risk factors.

**Results:**

Baseline SRH was reported as excellent by 17.8% participants, good by 65.1%, fair by 16.0% and poor by 1.2%. During 225,508 person-years of follow-up, there were 55 (21.2%) CVD events in the poor SRH group and 259 (7.0%) in the excellent SRH group (HR 3.7, 95% CI 2.8–4.9). The HR remained significant after adjustment for behavioural risk factors (HR 2.6, 95% CI 1.9–3.5) and after adjustment for all socio-demographic, clinical and behavioural risk factors (HR 3.3, 95% CI 2.4–4.4). Associations were strong for both fatal and non-fatal events and remained strong over time.

**Conclusions:**

SRH is a strong predictor of incident fatal and non-fatal CVD events in this healthy, middle-aged population. Some of the association is explained by lifestyle behaviours, but SRH remains a strong predictor after adjustment for socio-demographic, clinical and behavioural risk factors and after a decade of follow-up. This easily accessible patient-centred measure of health status may be a useful indicator of individual and population health for those working in primary care and public health.

## Introduction

Self-rated health (SRH), assessed through a single question, is strongly and consistently associated with all-cause mortality [1] and with mortality and morbidity due to a range of chronic diseases including diabetes, heart disease, stroke and cancer [2]–[7]. However interpretation of this finding has been hampered by the possibility of reverse causation and residual confounding due to poor characterisation of the populations studied, both in terms of prevalent disease status and of hypothesised mediators including health related behaviours [8]. We hypothesised that SRH will be strongly and robustly related to incident cardiovascular disease (CVD) events in a population without prevalent disease, that the relationship will remain strong over time, and that the relationship will be at least partially explained by health-related behaviours including smoking, physical activity and diet [8], [9].

We report incidence of first CVD event (fatal and non-fatal) by self-rated health status over a mean of 11 years in a population-based cohort of healthy 39 to 74 year olds without prevalent CVD recruited from general practices in Eastern England.

## Methods

### Ethics Statement

The European Prospective Investigation of Cancer (EPIC)-Norfolk study was approved by the Norfolk Local Research Ethics Committee and participants gave written consent prior to the first health check.

### Participants

EPIC-Norfolk is a prospective cohort study which recruited men and women aged 39–74 years from general practices in the Norfolk region of the UK [10]. In brief, between 1993 and 1997, 77,630 individuals were invited to take part in the study. Of these, 25,639 (33%) consented and attended a baseline health examination. This included anthropometric, venesection and blood pressure measurements and completion of validated questionnaires [11], [12].

### Self-rated Health and Covariate Measurement

SRH was measured using a single question: “In general, would you say your health is?” with response options: excellent, good, moderate or poor. Socio-demographic covariates included age, sex and education. Participants’ educational status was based on the highest qualification attained from the baseline questionnaire. It was categorised into four groups: degree or equivalent, A-level or equivalent, O-level or equivalent and less than O-level or no qualifications. O-level or equivalent indicates educational attainment to the usual minimal school leaving age of 15/16 and A-level to the age of 17/18 years. Occupational social class classified according to the Registrar General’s occupation based scheme was dichotomised into manual (skilled, partly skilled and unskilled manual workers) versus non-manual (professionals, managerial and technical occupations and non-manual skilled workers). Behavioural covariates included smoking, alcohol use, vitamin C intake and physical activity. Smoking behaviour was measured with the questions “Have you ever smoked as much as one cigarette a day for as long as a year?” and “Do you smoke cigarettes now?” and was classified into “Current smoker” and “Never smoked or former smoker”. Alcohol consumption was derived from the question “How many alcoholic drinks do you have each week?” with four separate categories of drinks. A unit of alcohol (about 8 g) was defined as a half pint (about 0.2 L) of beer, cider or lager; a glass of wine; a single unit of spirits (whisky, gin, brandy or vodka); or a glass of sherry, port, vermouth or liqueurs. Total alcohol consumption was estimated as the total units of drinks consumed in a week and dichotomised as moderate drinker (defined as one or more units a week but not more than 14 units a week) versus non-drinker/heavy drinker. Fruit and vegetable intake was estimated from plasma vitamin C levels. A blood-plasma value of ≥50 µmol/L vitamin C indicates an intake of at least 5 servings of fruit and vegetables daily. Plasma was stabilised in a standardised volume of metaphosphoric acid stored at −70°C and vitamin C concentration estimated using a fluorometric assay within 1 week of sampling [13]. The coefficient of variation was 5.6% at the lower end of the range (mean, 33.2 lmol/l) and 4.6% at the upper end (mean, 102.3 lmol/l). Physical activity was estimated from a validated self-reported questionnaire [11], with questions about work and leisure time activity during the past year. Individuals were defined as physically active if they had a sedentary occupation but reported at least 30 minutes leisure time activity a day, e.g. cycling or swimming; or else a non-sedentary occupation with or without leisure time activity. Clinical risk factors included measures of total cholesterol, systolic blood pressure, BMI, history of diabetes and family history of CVD. At baseline, trained nurses carried out a health examination to measure risk factors for disease. Height and weight were measured with participants in light clothing and the body mass index was calculated (weight (kg)/height (m)^2^). Blood pressure was measured using an Accutorr sphygmomanometer after each participant had been seated for 5 minutes. We used the mean of two measurements of blood pressure in analysis. At the baseline clinic visit nurses also took non-fasting blood samples. Serum concentrations of total cholesterol, high density lipoprotein (HDL) cholesterol and triglycerides were measured. Participants were asked if they had ever been diagnosed with diabetes mellitus by a doctor and if they had a family history of myocardial infarction or stroke. Data were missing in fewer than 2% of individuals for each covariate and were assumed to be missing at random. No multiple imputation was used. EPIC-Norfolk participants are comparable to contemporaneous UK population samples with respect to anthropometry, blood pressure and lipids, with fewer current smokers [10].

### Outcome Measurement

We report the results of follow-up to March 31^st^ 2007, a mean of 11 years. All EPIC-Norfolk participants were flagged for mortality at the UK Office of National Statistics. Death certificates were expertly coded using the International Classification of Diseases (ICD, revisions 9 and 10).

Hospital admissions were identified from participants’ National Health Service number by data linkage with the East Norfolk Health Authority database (ENCORE). This identifies hospital contacts throughout England and Wales for Norfolk residents. Participants were identified as having a CVD event during follow-up if CVD was the underlying cause of a hospital admission or death. CVD was defined using codes ICD-9-CM 410–414 or ICD-10 I20–I25 or ICD-9-CM 430–438 or ICD-10 I60–69. Codes included unstable and stable angina, myocardial infarction, subarachnoid and intracerebral haemorrhage and cerebral infarction. Validation studies indicate high specificity for case ascertainment [14].

### Statistical Analyses

We excluded individuals (i) who reported previous stroke, heart attack or angina (n = 1,932), (ii) those with missing SRH data (n = 273), and (iii) those with missing CVD risk factor data (cholesterol, blood pressure, history of diabetes, or smoking status (n = 2,576)). The study population therefore comprised 9,087 men and 11,827 women for analysis.

We summarised baseline socio-demographic, behavioural and clinical characteristics, and CVD outcomes, by self-rated health categories (poor/fair/good/excellent). We conducted a Cox regression analysis to investigate the crude and adjusted hazard ratios for CVD incidence by baseline SRH category after testing the proportional hazard assumption using the log-cumulative hazard plot and by comparing the predicted survival plot to the Kaplan-Meier plot. Models were block adjusted by socio-demographic variables (age, sex, and education); behavioural risk factors (smoking, alcohol intake, vitamin C intake and physical activity), and clinical variables (total cholesterol, systolic blood pressure, BMI, diagnosis of diabetes, and family history of myocardial infarction and stroke). In a final fully adjusted model, we adjusted for all blocks. As there was no significant interaction between SRH and sex in the association with CVD outcome (p-values Good SRH; 0.90, Moderate SRH; 0.10, Poor SRH; 0.95) we did not stratify the analyses by sex. To determine if the associations with CVD events persisted over time, Cox proportional hazards models with delayed entry were used. We distinguished three risk periods: short- (5 years following the baseline interview), mid- (between 5 and 10 years, after exclusion of CVD events which occurred in the first 5 years of follow-up), and long-term (between 10 and 14 years, after exclusion of the CVD events in the first 10 years). All analyses were performed using Stata version 11.0.

## Results

Baseline characteristics and CVD outcomes by SRH category are presented in Table 1. 17.8% (n = 3,712) participants reported excellent SRH, 65.1% (n = 13,604) good, 16.0% (n = 3,338) moderate and 1.2% (n = 260) poor health. The proportion of the population reporting low levels of education and the proportion of females increased across categories of SRH from excellent to poor. Individuals reporting poor SRH had the highest smoking rate, the highest alcohol consumption, the lowest vitamin C intake, and the lowest physical activity levels. Those with poor SRH also had elevated CVD risk factors and were more likely to be diagnosed with diabetes and to report a family history of stroke or myocardial infarction.

**Table 1:** Distribution of socio-demographic, behavioural and clinical risk factors for cardiovascular disease (CVD) and CVD outcomes by self-rated health (SRH) score in 9,087 men and 11,827 women aged 39–79 years without prevalent CVD in EPIC-Norfolk (1992–2007).

Variable	SRH Excellent	SRH Good	SRH Moderate	SRH Poor
	n = 3,712 (17.8%)	n = 13,604 (65.1%)	n = 3,338 (16.0%)	n = 260 (1.2%)
	Percent (n)/Mean (SD)			
**Socio-demographic**				
Age (years)	57.2 (9.0)	58.0 (9.3)	59.1 (9.3)	58.2 (8.9)
Female sex	52.9 (1,962)	56.8 (7,728)	59.3 (1,979)	60.8 (158)
Education				
*Less than O-level or no qualifications*	25.3 (940)	35.3 (4,797)	48.2 (1,610)	51.5 (134)
*O-level or equivalent*	10.2 (380)	10.9 (1,482)	8.8 (295)	11.5 (30)
*A-level or equivalent*	44.3 (1,645)	40.8 (5,551)	34.5 (1,151)	30.8 (80)
*Degree or equivalent*	20.1 (747)	13.0 (1,774)	8.5 (282)	6.2 (16)
**Behavioural**				
Current smoker	7.9 (294)	11.4 (1,550)	16.6 (554)	19.2 (50)
No alcohol use or more than 14 units/week	34.2 (1,270)	33.8 (4,601)	41.4 (1,381)	54.6 (142)
Vitamin C intake less than 5 servings of fruit and vegetables a day	31.9 (1,110)	37.9 (4,823)	47.3 (1,451)	56.1 (138)
Physically inactive (sedentary occupation and less than 30 minutes leisure time activity a day)	21.2 (787)	26.9 (3,656)	40.6 (1,356)	57.7 (150)
**Clinical**				
Total cholesterol (mmol/L)	6.1 (1.1)	6.1 (1.1)	6.2 (1.2)	6.2 (1.2)
Systolic blood pressure (mmHg)	132.5 (17.4)	134.9(18.2)	136.8 (18.9)	136.1 (18.2)
Diagnosis of diabetes	0.4 (14)	1.6 (216)	3.1 (103)	4.2 (11)
Body mass index (kg/m2)	25.5 (3.3)	26.1 (3.7)	26.9 (4.4)	27.4 (6.0)
Family history of myocardial infarction	34.0 (1,263)	35.5 (4,823)	37.4 (1,247)	41.9 (109)
Family history of stroke	21.9 (813)	24.4 (3,314)	24.5 (817)	25.0 (65)
**CVD outcomes**				
No event	3,453 (93.0)	12,251 (90.1)	2,829 (84.8)	205 (78.9)
CVD event	259 (7.0)	1,353 (10.0)	509 (15.3)	55 (21.2)
*Non-fatal CVD events*	*237 (6.4)*	*1,181 (8.7)*	*440 (13.2)*	*52 (20.0)*
Hospitalisation for ischemicheart disease	182 (4.9)	893 (6.6)	337 (10.1)	41 (15.8)
Hospitalisation for stroke	55 (1.5)	288 (2.1)	103 (3.1)	11 (4.2)
*Fatal CVD events*	*22 (0.6)*	*172 (1.3)*	*69 (2.1)*	*3 (1.2)*
Mortality for ischemic heartdisease	15 (0.4)	134 (1.0)	55 (1.7)	3 (1.2)
Mortality for stroke	7 (0.2)	38 (0.3)	14 (0.4)	0 (0.0)

Over a mean of 11 years (range 0–14 years, 225.508 person-years of follow-up), there were 2,176 incident CVD events. The cumulative incidence rate of CVD was 9.6 per 1,000 person-years. Incident events increased in frequency from excellent to poor SRH. There were 259 (7.0%) events in the excellent SRH group, 1,353 (10.0%) events in the good SRH group, 509 (15.3%) events in the moderate SRH group, and 55 (21.2%) CVD events in the poor SRH group.

**Figure 1:**
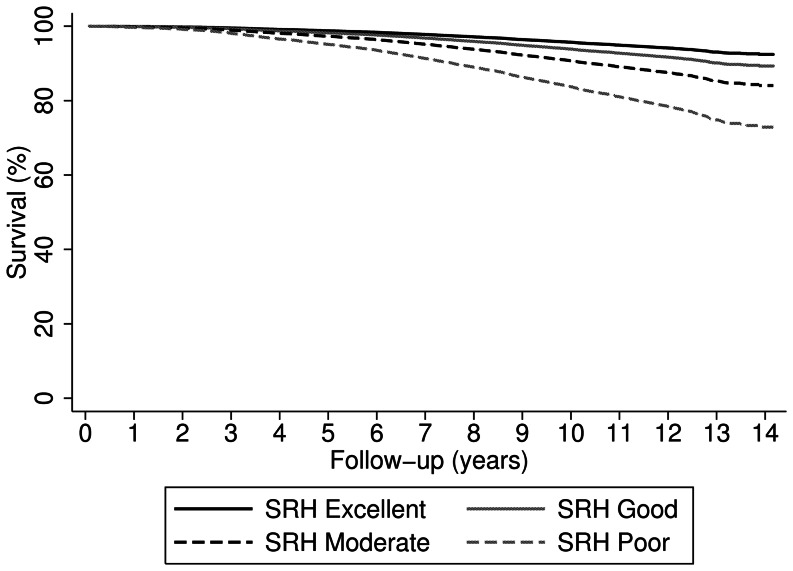
Survival curve for cardiovascular disease events by self-rated health score. Survival curve for the hospitalisation and mortality from ischemic heart disease or stroke by self-rated health (SRH) score in 7,279 men and 9,285 women aged 39–79 without previous cardiovascular disease in EPIC-Norfolk (1992–2007), fully adjusted for sociodemographic variables (age, sex and education), behavioural risk factors (smoking, alcohol use, vitamin C intake and physical activity) and clinical risk factors (total cholesterol, systolic blood pressure, BMI, history of diabetes and family history of myocardial infarction or stroke).

The proportional hazard assumption was not violated. Table 2 shows the hazard ratio (HR) of a fatal or non-fatal CVD event for SRH, adjusting for different blocks of possible confounders. Compared with excellent SRH, the risk of a CVD event increased across categories of worsening SRH (good: HR = 1.5 (95% CI 1.3–1.7); moderate: HR = 2.4 (95% CI 2.0–2.8); poor: HR = 3.7 (95% CI 2.8–4.9)) in crude analysis. Results were similar for fatal and non-fatal CVD events, although only few fatal events occurred (n = 266, of which n = 3 in those with poor SRH). Adjustment for age, sex and education had little effect on HRs. No significant interaction was found between age or sex and SRH (data not shown). Adjusting for behavioural risk factors slightly attenuated the association between SRH and CVD events, with the HR of poor versus excellent SRH reducing from 3.7 (95% CI 2.8–4.9) to 2.6 (95% CI 1.9–3.5). Adjustment for clinical risk factors had little effect on the strength of the association. In the fully adjusted model, there remained a strong, independent effect of SRH on incident CVD events. Relative to those reporting excellent health, those reporting good (HR = 1.3; 95% CI 1.1–1.5); moderate (HR = 1.9; 95% CI 1.6–2.2); and poor SRH (HR = 3.3; 95% CI 2.4–4.4) had a higher risk of a CVD event (Figure 1). SRH was strongly associated with CVD events at short-, mid- and long-term follow-up (Table 3). The strength of the association diminished over time from 3.7 (95% CI 2.1–6.3) for poor versus excellent SRH in the first 5 years after baseline, to 3.2 (2.1–4.8) between 5 and 10 years follow-up and 2.3 (95% CI 1.1–4.9) between 10 and 14 years of follow-up.

**Table 2:** Hazard ratio of hospitalisation and mortality from ischemic heart disease and stroke for self-rated health (SRH) adjusted for socio-demographic variables, behavioural risk factors, clinical risk factors for cardiovascular disease (CVD) in 7,279 men and 9,285 women aged 39–79 years without prevalent CVD in EPIC-Norfolk (1992–2007).

Self-rated health category	Fatal and non-fatal CVD events	Non-fatal CVD events	Fatal CVD events
	Hospitalisation and mortality from ischemic heart disease and stroke	Hospitalisation from ischemic heart disease and stroke	Mortality from ischemic heart disease and stroke
	HR (95% CI)	HR (95% CI)	HR (95% CI)
**Model 1: Unadjusted**			
Excellent	Ref.	Ref.	Ref.
Good	1.5 (1.3–1.7)	1.4 (1.2–1.6)	2.2 (1.4–3.4)
Moderate	2.4 (2.0–2.8)	2.3 (1.9–2.6)	3.7 (2.3–6.0)
Poor	3.7 (2.8–4.9)	3.8 (2.8–5.2)	2.3 (0.7–7.6)*
**Model 2: Adjusted for socio-demographic risk factors** a			
Excellent	Ref.	Ref.	Ref.
Good	1.4 (1.2–1.7)	1.4 (1.2–1.6)	1.8 (1.2–2.9)
Moderate	2.3 (1.9–2.6)	2.2 (1.8–2.6)	2.9 (1.8–4.7)
Poor	3.9 (2.9–5.3)	4.1 (3.0–5.6)	2.2 (0.7–7.4)*
**Model 3: Adjusted for behavioural risk factors** b			
Excellent	Ref.	Ref.	Ref.
Good	1.4 (1.2–1.6)	1.3 (1.1–1.5)	1.8 (1.1–2.8)
Moderate	1.9 (1.6–2.3)	1.9 (1.6–2.2)	2.3 (1.4–3.8)
Poor	2.6 (1.9–3.5)	2.8 (2.1–3.9)	1.2 (0.3–3.9)*
**Model 4: Adjusted for clinical risk factors** c			
Excellent	Ref.	Ref.	Ref.
Good	1.4 (1.2–1.5)	1.3 (1.1–1.5)	2.0 (1.3–3.1)
Moderate	2.1 (1.8–2.4)	1.9 (1.7–2.3)	3.2 (2.0–5.2)
Poor	3.2 (2.4–4.3)	3.4 (2.5–4.5)	1.9 (0.6–6.5)*
**Model 5: Fully adjusted for socio-demographic, behavioural and clinical risk factors**			
Excellent	Ref.	Ref.	Ref.
Good	1.3 (1.1–1.5)	1.3 (1.1–1.5)	1.5 (0.9–2.4)
Moderate	1.9 (1.6–2.2)	1.9 (1.6–2.3)	2.1 (1.2–3.4)
Poor	3.3 (2.4–4.4)	3.5 (2.6–4.9)	1.5 (0.5–5.2)*

a **Socio-demographic risk factors**: Age, sex and education.

b **Behavioural risk factors:** Smoking, alcohol use, vitamin C intake and physical activity.

c **Clinical risk factors:** Total cholesterol, systolic blood pressure, BMI, history of diabetes and family history of myocardial infarction or stroke.

* **Based on small numbers: n = 3 of n = 260 participants with poor SRH had a fatal CVD event during follow-up.**

**Table 3:** Hazard ratios of hospitalisation and mortality from stroke and ischemic heart disease for self-rated health (SRH) at short-, mid- and long-term, cox models with delayed entry.

Variable	Hospitalisation and mortality from ischemic heart disease or stroke
	HR (95% CI)
***Short-term risk (0–5 years)***	
*N = 20,914 (n = 546 CVD events)*	
**SRH**	
Excellent	Ref.
Good	1.4 (1.1–1.9)
Moderate	2.2 (1.6–3.0)
Poor	3.7 (2.1–6.3)
***Mid-term risk (5–10 years)***	
*N = 19,926 (n = 1168 CVD events)*	
**SRH**	
Excellent	Ref.
Good	1.4 (1.1–1.6)
Moderate	1.9 (1.5–2.3)
Poor	3.2 (2.1–4.8)
***Long-term risk (10–15 years)***	
*N = 15,457 (n = 462 CVD events)*	
**SRH**	
Excellent	Ref.
Good	1.1 (0.8–1.4)
Moderate	1.6 (1.2–2.2)
Poor	2.3 (1.1–4.9)

Each of the models was fully adjusted for sociodemographic variables (age, sex and education), behavioural risk factors (smoking, alcohol use, vitamin C intake and physical activity) and clinical risk factors (total cholesterol, systolic blood pressure, BMI, history of diabetes and family history of myocardial infarction or stroke).

See table 2 for the risk over the total follow-up period (0–14 years).

## Discussion

We have shown that SRH is a robust predictor of incident fatal and non-fatal CVD events over 11 years in this large, well characterised, middle-aged population in Eastern England. The relationship between SRH and CVD was explained in part by behavioural risk factors, but a strong independent effect remains after full adjustment for socio-demographic, behavioural, and clinical risk factors.

### Strengths

This study is among the largest of the prospective studies [6], [15], [16] with long follow-up [2], [3], [5], [6], [15], [17], [18]. Inclusion of men and women of middle age [2], [15], [19] complements and extends previous studies which have included different populations and shown weaker associations in younger people and men [2]–[6], [15]–[20]. For example a large Finnish study with long follow-up (23 years) investigated the relationship between SRH and CVD mortality in a younger cohort (mean age 42–43 years, range 25–64) [6]. After adjustment for baseline CVD, age, education, survey year, cholesterol, blood pressure, BMI and two behavioural variables (self-reported smoking status and leisure time physical activity), they found a smaller independent effect of SRH (SRH poor vs. good: men 1.7 (1.5–1.9); women 1.6 (1.3–2.1)). Another Finnish cohort that included fatal and non-fatal CHD outcomes was restricted to 2,512 middle-aged men and 6 year follow-up and assessed smoking, alcohol and leisure time physical activity by self-report [4]. Those with poor SRH compared to good SRH were 2.1 times more likely to die from CVD and 1.5 times more likely to have a fatal or non-fatal myocardial infarction, independent of age, income and behavioural and clinical risk factors.

We made particular efforts to exclude people with subclinical disease, and the strong associations that we found, that persisted over a decade of follow-up, are therefore of particular interest. Previous studies have not excluded prevalent disease, or have included older cohorts where results are more likely to reflect current ill health [3]–[6]. We excluded people reporting a diagnosis of stroke, heart attack or angina, and adjusted for cardiovascular risk at baseline. Precise characterisation of factors that might affect the relationship between SRH and CVD, including health behaviours and clinical risk factors, is often a challenge in epidemiological studies. In EPIC-Norfolk, the baseline health examination was carried out by trained nurses using standard operating procedures and included validated questionnaires. We also used an objective measure of fruit and vegetable intake (plasma vitamin C). Our results thus extend previous studies that have measured fewer health-related behaviours, have used self-report of dietary intake, or have not accounted for the positive effects of moderate alcohol consumption on coronary heart disease compared with negative effects on cardiovascular health of alcohol abstention as well as higher consumption [21]. Outcomes were robustly defined and measured.

### Limitations

Although the response rate was 33%, sample characteristics were comparable to contemporaneous UK population samples other than smoking [10]. Results from this population-based study are thus likely to be usefully applicable to men and women aged 39–79 years, who are in the age range targeted for preventive action for example in the UK Health check project (University of Leicester).

We tried hard to limit reverse causation. We excluded people reporting a diagnosis of stroke, heart attack or angina, and adjusted for cardiovascular risk at baseline. Moreover, a sensitivity analysis excluding those with high CVD risk (Framingham risk score of 20% or higher) showed similar results (Good SRH: HR = 1.5 (95% CI 1.1–1.8); Moderate SRH: HR = 2.3 (95% CI 1.8–3.1); Poor SRH: HR = 3.6 (95% CI 2.2–5.8), fully adjusted model). The lack of association between cardiovascular risk and SRH at baseline alongside the enduring relationship over more than 10 years must make reverse causation an unlikely explanation for our findings. However, some effect of subclinical disease on SRH (reverse causation) remains possible, and this is supported by the stronger relationship between SRH and CVD observed over a shorter follow-up time.

We cannot exclude the possibility of an effect of residual confounding. We did not investigate the effects of psychosocial factors, which have been linked with mortality in those with poor SRH [22]–[24]. However, in at least one other study, factors such as work absence, stress and personal and financial problems were not significantly associated with CVD hospitalisation and only slightly attenuated the relationships between SRH and CVD events [2]. We could not assess disease severity, although in the recent WISE study among women at risk of ischaemic heart disease, severity of disease assessed by quantitative analysis of coronary angiograms did not explain the association between SRH and outcomes [25]. Use of hospital admission data may underestimate non-fatal CVD events that did not lead to hospital admission. Social class is associated with both SRH and CVD events [15], [26]–[28] and although we adjusted for education, we cannot exclude residual confounding from social class. However, we have previously shown that there is no significant effect modification between SRH and all-cause mortality or CVD events by social class [29] and a sensitivity analysis adjusting for occupational social class showed similar results to the analysis adjusting for education (Good SRH: HR = 1.4 (95% CI 1.2–1.6); Moderate SRH: HR = 2.2 (95% CI 1.9–2.6); Poor SRH; HR = 3.9 (95% CI 2.9–5.3), adjusted for age, sex and occupational social class).

While our results suggest that part of the association between SRH and CVD outcomes is independent of health behaviour, we cannot exclude collider bias. Aside from fruit and vegetable intake, health-related behaviours were self-reported, which can lead to overestimation, and may have diluted the true association with CVD outcomes [30].

Only 260 participants reported their health as poor and 55 CVD events occurred in this group. This reduction in power may have influenced results and made multiple confounder adjustment less efficient. However, using a categorical measure of SRH captured the graded relationship between decreasing SRH and CVD.

### Mechanisms

Clinical risk factors have a strong relationship with SRH in studies that have not excluded prevalent disease [1], [31], but accounted for little of the relationship between SRH and CVD in our analyses among healthy people. The protective relationship between healthy behaviours and CVD incidence is well established [8], [21], [32]. In our data, behaviours were associated with SRH at baseline, and accounted for part of the excess hazard of poor versus excellent SRH for incident CVD events, but the association between SRH and CVD remained strongly significant after adjustment for behaviours. Where other studies have reported that part of the relationship between SRH and CVD events is explained by behavioural and clinical risk factors, they have not used objective measures of diet or adjusted separately for these factors [4]–[6]. However, further exploration of individual behaviours showed no difference in impact between self-reported and objectively measured behaviours (data not shown). To further understand how SRH and health behaviours are associated with CVD outcomes more complex analyses are needed.

Some have conceptualised SRH as a stable characteristic indicating a person’s established beliefs about his health [33]. It has been suggested that more optimistic people rate their health more positively and that it is this underlying predisposition that is driving the association with outcome [34]. SRH could also conceivably directly affect immune responses or neuro-endocrine homeostasis, although there are few data yet to support this [35]. The problems of assessment of disease prevalence, severity and of residual confounding limit the contribution of prospective epidemiological studies alone in investigating the mechanisms underlying the relationship between SRH and CVD. Novel characterisation of possible mediators may take the field forward, for example by hypothesised neuroendocrine factors or by assessment of health awareness, positive attitudes and self-care. These will require work both in the laboratory and qualitative study in the field to define and characterise likely modifiable determinants. Advances in understanding mechanism will also depend on randomised trials of interventions to change these hypothesised determinants. Qualitative study of potential determinants of SRH may be particularly informative for developing potential interventions before studying their effects on CVD incidence.

### Clinical Implications

Risk models based on conventional risk factors fail to correctly categorise up to a quarter of CVD risk [36]. Risk scoring has been dominated by the accuracy of model prediction and not by feasibility of application in primary care, where most primary prevention takes place. Attempts to improve on performance in the clinic can plausibly lead in two directions. Firstly towards more complex risk scores including novel blood or urine bio-markers (such as C-reactive protein (CRP) and N-terminal pro-brain natriuretic peptide (NT-proBNP)), genetic markers or non-invasive imaging (such as carotid intima-media thickness). Secondly towards simplified strategies of risk assessment to engage high risk communities who might benefit most from screening in primary care. Our work adds to the evidence that SRH has a place in the second strategy. SRH is an easy to collect patient-centred assessment of health risk that is meaningful both to the patient and practitioner. It has been shown to have high summary item validity, reproducibility and reliability and a consistency of association with health outcomes including cardiovascular health regardless of age, geographical region and language [1], [37]–[40]. Moreover, preliminary evidence suggests that it can form part of a simple non-invasive risk score that out-performs the established Framingham score in specific populations [41]. Used alone, or with other risk factors, it may be a simple and useful indicator for individual and population cardiovascular health for those working in primary care and public health in any global setting. For example, it could be used to identify groups of patients at particular risk of cardiovascular disease and to target health promotion and health policy strategies towards such groups. SRH could also be potentially used as an outcome measure for assessing any such strategies or interventions. It is also conceivable that interventions targeted at improving SRH itself may be used to improve cardiovascular health and disease related outcomes.

### Conclusions

SRH is a strong predictor of incident fatal and non-fatal CVD events in this healthy, middle-aged population over a decade. Some of the association is explained by lifestyle behaviours, but SRH remains a strong predictor after adjustment for socio-demographic, clinical and behavioural risk factors. This easily accessible patient-centred measure of health status may be a useful indicator of individual and population health for those working in primary care and public health and demands more research on its place in preventive medicine.
